# Single-unit activity in the anterior claustrum during memory retrieval after trace fear conditioning

**DOI:** 10.1371/journal.pone.0318307

**Published:** 2025-02-11

**Authors:** Sewon Park, Kuenbae Sohn, Donghyeon Yoon, Junghwa Lee, Sukwoo Choi

**Affiliations:** 1 Department of Neurobiology, University of Utah, Salt Lake City, Utah, United States of America; 2 School of Biological Sciences, College of Natural Sciences, Seoul National University, Seoul, Korea; Guizhou University of Traditional Chinese Medicine, CHINA

## Abstract

We have recently identified a group of claustral neurons that continuously maintain information associated with a fear-conditioned stimulus (CS) for at least tens of seconds, even after the CS has ceased. This “online state” refers to the persistent maintenance of threat-associated information, enabling it to be actively processed even after the threat has terminated. This state may involve reciprocal interactions of the claustral neurons with brain regions involved in decision-making, motor preparation, and adaptive behavioral responses. If these claustral neurons truly encode the online state, their function should remain independent of the modality of the threat stimulus or the specific defensive behavior exhibited. In this study, we used a tone cue and monitored freezing behavior in trace conditioning, in contrast to the light cue and escape behavior used in our recent study. During the retrieval test of trace conditioning, a subset of rostral-to-striatum claustrum (rsCla) neurons exhibited sustained activity in response to the CS, particularly during the trace interval. Importantly, we found a positive correlation between the activity of rsCla neurons and the magnitude of freezing during the trace interval, when intervals without freezing were excluded. Thus, this subset of rsCla neurons appears to exhibit the characteristics of ‘online neurons’ during memory retrieval following trace conditioning.

## Introduction

Instrumental behaviors are learned via direct experience of the action-outcome relationship; for instance, the animals must learn that lever pressing produces food or shuttling avoids harm [1,2]. The direct experience of the action-outcome relationship is thought to provide value to the action in a particular situation, which therefore allows the animals to choose adequate goal-directed behaviors when encountering the same or similar situations [3,4]. Recently, it has been proposed that non-human animals may go beyond their direct experience of the action-outcome relationship; that is, they may show deliberative defensive behaviors guided by constructive or extrapolative planning, in which the ultimate outcome of their actions can be forecasted in the absence of prior experience [1]. Consistent with this prediction, we have recently established a new behavioral task called ‘delayed escape behavior’ in which the subject shows faster escape responses to a potentially threatening stimulus compared to controls without direct experience of the outcome of the escape [5]. In the case of active avoidance typically conducted in a shuttling box following CS-US pairing, the escape response must occur during the presentation of the CS, and failure to do so results in a shock. This type of behavior test requires repeated experiences of the consequences to learn the avoidance behavior. However, in the delayed escape test used in this study, following CS-US pairing, the rat is first exposed to the CS and must infer that an adjacent space, rather than the space where the CS is presented, would be a relatively safer option. The task meets the following conditions: 1) the location to escape to should not be perceived as a safe shelter to exclude the possibility of innate escape, 2) the escape behavior should not be associated with nor reinforced by its outcome to rule out the possibility of learned escape, 3) the escape behavior should be executed after a delay period following the threat stimulus, and neural activity encoding this delay period must be observed, since there exists a delay between stimulus and execution due to cognitive processing in the case of behaviors mediated by higher cognitive functions. Therefore, the last premise enables the prediction that escape behavior requires persistent neural activity to hold information for generating the escape behavior. Indeed, we have found such a persistently enhanced population activity in a subset of rostral-to-striatum claustrum (rsCla) neurons and their transient inhibition during the early phase of the delay attenuates both the persistent activity and delayed escape behavior, suggesting that recurrent activity underlies the persistent activity, and that the persistent activity is required for delayed escape behavior. Since the claustrum has been proposed as a hub for multiple brain regions of high cognitive functions, the persistent activity in the rsCla may represent that multiple brain regions involved in the delayed escape behavior (e.g., the prefrontal cortex, orbitofrontal cortex and hippocampus) have simultaneously entered an online state.

The proposed function of rsCla neurons in the present study along with our recent publication [5] contrasts with previous findings on the claustrum and may, in fact, align with the traditional role of the claustrum as a central hub of consciousness, as originally suggested by Dr. Francis Crick [6]. It has been reported in previous studies that the claustrum serves various functions such as attention [7–12], cortical slow-wave coordination [13,14], associative learning [15,16], memory consolidation [17], anxiety responses [18], motor preparation [19], behavioral engagement [20], spatial processing [21–23], and cognitive conflict tasks [24]. Thus, the function of the claustrum appears to be multifaceted, and based on these findings, it may be difficult to define its function with a single description. Anatomically, the claustrum is a thin, elongated region, and its function could be divided among its various subregions. Notably, studies on rsCla, which is the focus of this research, remain relatively sparse (see [23]). Previous studies suggest that the anterior portion of the claustrum interacts more strongly with the frontal cortices, indicating that rsCla might have a specific function, one of which could be the maintenance of the online state.

The concept of ‘online neurons’ is distinct from attention, arousal, and motor preparation, as demonstrated in our recent study on ‘delayed escape behavior’ wherein the subject escapes from one compartment to the other via a small outlet [5]. ‘Online neurons’ emphasize the sustained maintenance of threat-related information even after the conditioned stimulus (CS) has ended. In our recent study, we have not observed any changes in attention, arousal, or motor preparation following the inhibition of rsCla neurons [5]. This conclusion is supported by the absence of alterations in reaction times of head angle adjustment in response to the opening of the escape door, as well as the speed of body movement on the floor after optogenetic inhibition of rsCla neurons. Furthermore, there were no instances where the rats crossed the outlet in a single attempt. Instead, they typically engaged in exploratory behavior toward the other compartment by placing their forepaws on the outlet ledge and then returning to the floor (the escape outlet is positioned at a height that allows the rats to place their front paws on the ledge while keeping their hind feet on the ground). This “forepaws-on” behavior was repeated an average of three times before the rats ultimately crossed. Notably, this behavior occurred independently of the timing of the escape, and the persistent activity of rsCla neurons decreased during the forepaws-on behavior. Therefore, it is challenging to associate the persistently enhanced activity of rsCla neurons with motor preparation.

Classical conditioning has long served as a foundational model in facilitating in-depth studies into various brain functions such as behavior, memory, and even high cognition [15,25–27]. Classical conditioning can be divided into two main types: delayed conditioning, where the CS and the brief unconditioned stimulus (US) are co-terminated, and trace conditioning, where there is a temporal gap between the CS and US. In delayed conditioning, the CS and the US are presented simultaneously, which facilitates contingency learning between the two stimuli. However, in trace conditioning, there is an interval (trace interval) between the presentation of the CS and the US, requiring the neural representation of the CS to be maintained until the US is presented for contingency learning to occur. Therefore, trace conditioning is considered more cognitively demanding than delayed conditioning [28,29]. Indeed, it has been shown to involve brain regions associated with higher cognitive functions such as the prefrontal cortex and hippocampus, and persistent activity has been observed in the prefrontal cortex during the trace interval [30–32].

In the present study, we aimed to identify a subset of rsCla neurons that exhibit the characteristics of ‘online neurons’ in trace conditioning. While our recent study [5] investigated rsCla activity using a light CS and escape behavior as outputs, the present study examined freezing behavior in response to a tone CS. Despite these differences in CS modality and monitored behavior, we observed persistent activity in a subset of rsCla neurons during the trace interval between the CS and US. This activity correlated positively with freezing behavior during the trace interval. These findings suggest that the persistent activity of rsCla neurons is independent of both the sensory modality of the CS and the type of behavioral response. This meets a key criterion for online neurons: their activity must remain modality- and behavior-independent, reflecting a general role in maintaining threat-related information across diverse conditions.

## Results

In our recent study [5], we observed that the online state is maintained even after a potentially threatening stimulus has disappeared. Based on this observation, we hypothesized that the period following the end of the conditioned stimulus (CS) in trace conditioning could be used to study the online state. Specifically, we considered the trace interval—the time between the end of the CS and the presentation of the unconditioned stimulus (US)—as an optimal period to observe the online state. Therefore, we have hypothesized that persistent activity in the rsCla also underlies an online state during the trace interval when the subject is predicting the occurrence of the US. To test this hypothesis, *in vivo* single unit recordings were conducted in the retrieval phase using rats with nichrome electrodes implanted in the rsCla. For the control group, all procedures were identical to those in trace conditioning, except that the subject underwent delayed conditioning for the acquisition phase.

**Fig 1:**
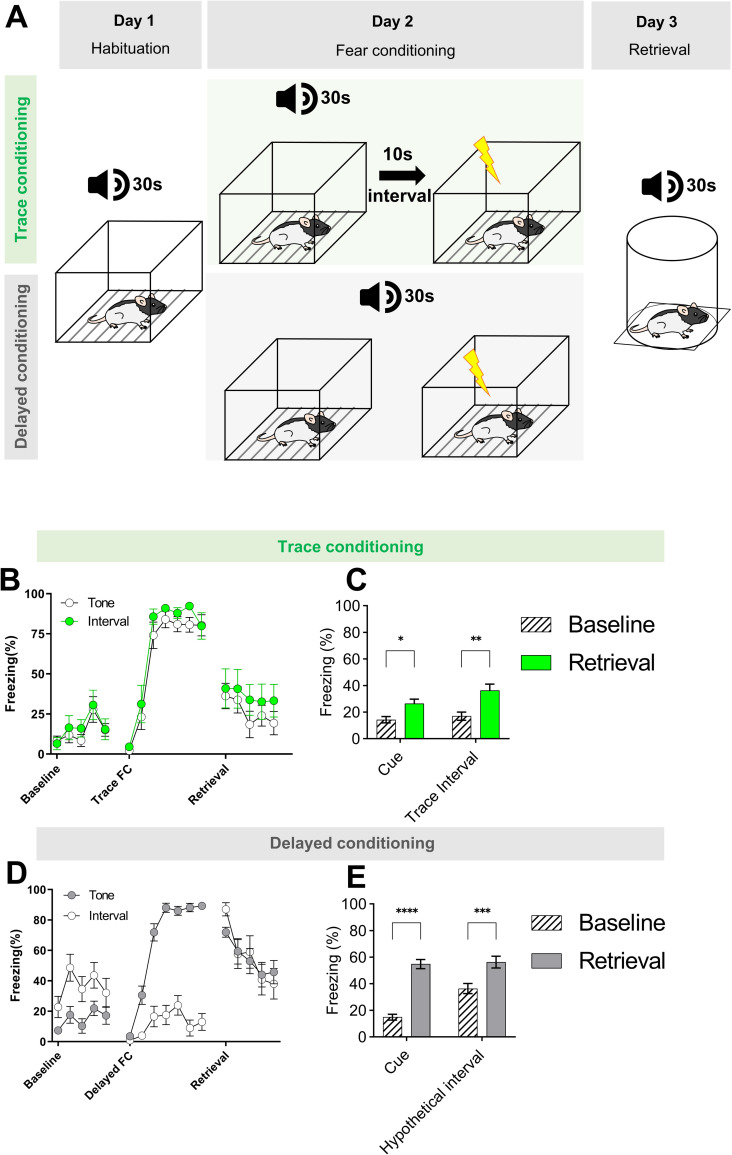
Trace and delayed conditioning. (A) Schematic illustration of trace conditioning and delayed conditioning. (B) A graph showing the percentage of freezing over time during trace conditioning. (n =  12). Open circles represent the percentage of freezing during the CS presentation, while filled circles represent the percentage of freezing during the trace interval. (C) A bar graph comparing the averaged percentage of freezing during the baseline period and the retrieval period, divided into the CS presentation period and the trace interval. During both the baseline and retrieval periods, the CS was presented five times. Statistical comparisons using Multiple Mann–Whitney tests followed by the Holm–Sidak method show significant differences: for CS presentation, * p =  0.025125; for trace intervals, **p  =  0.002557. Error bars represent s.e.m. (D) A graph showing the percentage of freezing over time during delayed conditioning (n =  17). Filled circles represent the percentage of freezing during the CS presentation, while open circles represent the percentage of freezing during the hypothetical trace interval. (E) A bar graph comparing the averaged percentage of freezing during the baseline period and the retrieval period, divided into the CS presentation period and the hypothetical trace interval. During both the baseline and retrieval periods, the CS was presented five times. Statistical comparisons using Multiple Mann–Whitney tests followed by the Holm–Sidak method show significant differences: for CS presentation, ****p <  0.0001; for hypothetical trace intervals, ***p =  0.000768. Error bars represent s.e.m.

Trace fear conditioning was conducted using the following procedure: Long Evans rats, 8 weeks old, underwent seven pairings of a 30-second CS and a 1-second US at average intervals of 200 seconds. The trace interval between the CS and US was 10 seconds. In contrast, delayed conditioning for the control group was performed similarly, but with a 30-second CS and a 1-second US paired to terminate simultaneously. After the acquisition phase, fear memory retrieval was conducted the following day with five CS presentations given at average intervals of 90 seconds. For the control group, as in trace conditioning, a hypothetical 10-second trace interval was set after the end of the CS (Fig 1).

**Fig 2:**
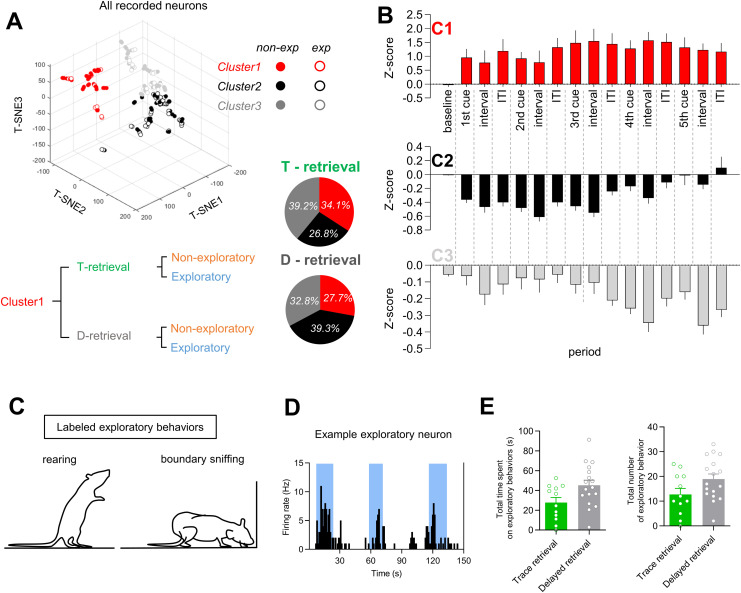
Clustering of claustral neurons based on behavioral epochs. (A) Distribution of clustered neurons (n = 173) in t-SNE space (top left). A schematic illustrating the process of subgroup division (bottom). Percentage of three clusters in the trace conditioning retrieval or delayed conditioning retrieval group (bottom right). The recorded number of cells per animal was 5.96 ±  4.4 units. Filled circles represent non-exploratory neurons, while open circles represent exploratory neurons. (B) *Z* scored firing rates of neurons within each cluster. C1 (top, n =  44), C2 (middle, n =  71), and C3 (bottom, n =  58). (C) Schematic representation of exploratory behavior. (D) An exemplary exploratory neuron shows an increase in firing rate during exploratory behavior. The periods of exploratory behavior are indicated by cyan shading. (E) This graph shows the total time (left) and total count (right) of exploratory behavior measured during the baseline period before CS presentation. Error bars indicate the standard error of the mean (s.e.m.).

Seventy units in the rsCla (from 12 rats) were recorded during the retrieval phases of trace conditioning. Additionally, 103 units (from 17 rats) were recorded during the retrieval phase of delayed conditioning. Out of a total of 173 neurons, 153 neurons exhibited excitatory response, while the remaining 20 neurons were observed as putative interneurons (S3 Fig). Histological analysis confirmed that these units had been recorded via electrodes with tips located in the rsCla (S8 Fig). To classify neurons in the rsCla upon their temporal activity patterns [33,34] in a non-biased manner, we used t-SNE (t-distributed stochastic neighbor embedding) and a subsequent K-means clustering algorithm with the support of the gap statistic as used in our recent study [5,35] (see Methods; Fig 2A, S1 Fig). We clustered the whole recorded neurons based on all the individual epochs including five CS presentations (i.e., a period for CS presentation, an interval from CS offset to US onset, inter-trial interval (ITI) from US offset to next CS onset). In the case of the control group, the trace interval was replaced with a 10-second period from the end of the CS (hypothetical trace interval). Neurons from the retrieval phases of both trace conditioning and delayed conditioning, were used for clustering. All the neurons from the two groups were clustered into three distinct clusters. Neurons in cluster 1 showed an enhanced activity which persisted from the first CS (Fig 2B, S4 Fig). For Cluster 2 neurons, they initially exhibited a depressed response, followed by a gradual recovery to baseline (Fig 2B, S7 Fig). In Cluster 3, there appeared to be an inhibitory pattern that progressively strengthened over time (Fig 2B, S7 Fig). Each cluster was subdivided into exploratory neurons and non-exploratory neurons (exploratory neurons were randomly distributed in the three clusters) (Fig 2C–E). Exploratory neurons refer to those that exhibit activity selectively associated with exploratory behavior (see more details in Methods). In contrast, non-exploratory neurons appear to be defined simply as neurons that are not selective to exploratory behavior. We excluded exploratory neurons for further analyses as shown in our recent study (see [5]), and focused on non-exploratory neurons of the three clusters. To validate the clustering results, we performed clustering exclusively on non-exploratory neurons instead of all recorded neurons. We observed that the groups formed by clustering non-exploratory neurons were highly similar to those obtained by clustering all recorded neurons and subsequently removing exploratory neurons (S2 Fig).

**Fig 3:**
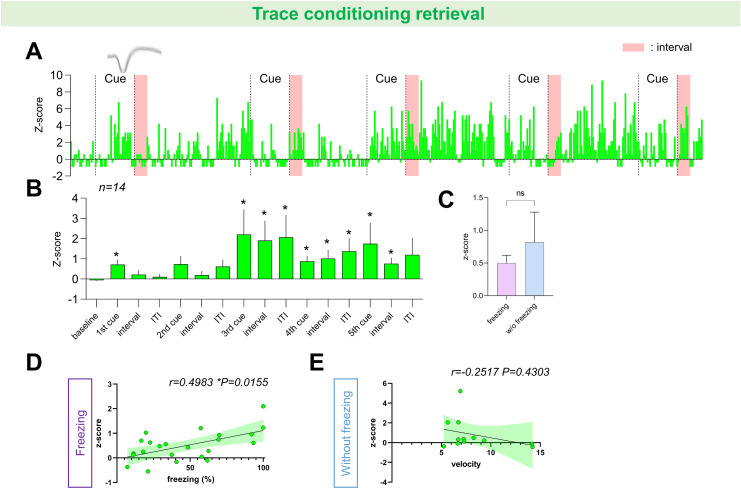
Non-Exploratory neurons in Cluster 1 during the retrieval phases of trace conditioning. (A) Representative *Z* score example of a non-exploratory neuron from the trace conditioning retrieval group in cluster 1, with example neuronal spike traces and cue presentation periods indicated by dashed lines. (B) The mean *Z* score values of non-exploratory neurons in cluster 1 of the trace conditioning retrieval group (n =  14) were found to change as the behavioral sessions progressed (**p =  0.0034; Friedman test followed by FDR by Benjamini and Hochberg, Q <  0.05 are marked with a star). (C) Comparison of average z-scores between trace intervals where freezing was observed and those where it was not (n = 23, 12 respectively; p =  0.5716 via Mann–Whitney tests). (D) Correlation between percent freezing and mean Z-scores during trace intervals where freezing was observed. Significant correlation was observed (n =  23; Spearman correlation r =  0.4983, * p =  0.0155). (E) Correlation between head velocity and mean Z-scores during trace intervals where freezing was not observed (n =  12; Spearman correlation r =  -0.2517, p =  0.4303). Solid lines and shades indicate linear regression and its 95% confidence intervals, respectively, for (D), (E).

**Fig 4:**
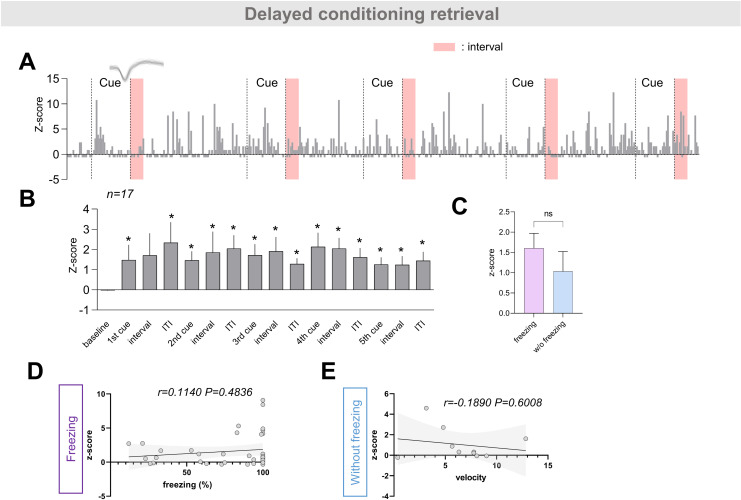
Non-exploratory neurons in Cluster 1 during the retrieval phases of delayed conditioning. (A) Representative *Z* score example of a non-exploratory neuron from the delayed conditioning retrieval group in cluster 1, with example neuronal spike traces and cue presentation periods indicated by dashed lines. (B) The mean *Z* score values of non-exploratory neurons in cluster 1 of the delayed conditioning retrieval group (n =  17) were found to change as the behavioral sessions progressed (****p <  0.0001; Friedman test followed by FDR by Benjamini and Hochberg, Q <  0.05 are marked with a star). (C) Comparison of average z-scores between trace intervals where freezing was observed and those where it was not (n = 40, 10 respectively; p =  0.5617 via Mann–Whitney tests). (D) Correlation between percent freezing and mean Z-scores during trace intervals where freezing was observed. Significant correlation was not observed (n =  40; Spearman correlation r =  0.1140, p =  0.4836). (E) Correlation between head velocity and mean Z-scores during trace intervals where freezing was not observed (n =  10; Spearman correlation r =  -0.1890, p =  0.6008). Solid lines and shades indicate linear regression and its 95% confidence intervals, respectively, for (D), (E).

In the three clusters of non-exploratory neurons, the basal firing rate ranged from 0.5 Hz to 4 Hz (S1 Table), and the three clusters of non-exploratory neurons included a small proportion of inhibitory neurons but the majority appeared to be excitatory neurons (S3 Fig). Since the neural activity in cluster 1 (non-exploratory neurons) exhibited persistency, a characteristic of ‘online neurons’ as shown in our recent study, further analyses were conducted using these neurons. Cluster 1 included non-exploratory neurons from two groups (trace conditioning retrieval, delayed conditioning retrieval), and we analyzed neurons from the two groups independently as shown in Figs 3 and 4.

One day after trace conditioning, memory retrieval was tested with five presentations of the CS. Statistically significant persistent activity was observed starting from the third CS (Fig 3). However, the freezing response showed a tendency to decrease across the five CS presentations (Fig 1). We first compared the average z-scores between intervals with and without observed freezing, finding no statistical difference between these periods. Nevertheless, a statistically significant positive correlation was found between the z-scores of individual units and percent freezing during intervals where freezing occurred (Fig 3D). As each rat received 5 CS presentations, 5 data points for the z-scores and 5 freezing values per rat were plotted. However, the data points were excluded if there was no freezing or if no neurons classified as cluster 1 were present during the given epoch. While there was a trend toward a positive correlation between the z-scores of individual units and percent freezing when all intervals were included, this was not statistically significant (S6 Fig). In contrast, no significant correlation was observed between the z-scores of individual units and percent freezing during either the cue presentation or the ITI. Taken together, the finding that persistent activity during the trace interval without freezing does not significantly differ from the persistent activity observed during the trace interval with freezing makes it difficult to support the hypothesis that persistent activity directly triggers freezing. Instead, it seems more appropriate to suggest that freezing during the trace interval is induced by other brain regions. Ultimately, the positive correlation between persistent activity and freezing during the trace interval is consistent with the hypothesis that the persistent activity of rsCla neurons has a modulatory effect on the already induced freezing behavior.

We lastly analyzed the control group consisting of a delayed conditioning retrieval group with the hypothetical trace interval period (Fig 4). Persistent activity began from the first ITI and persisted throughout the remaining period. There was no statistical difference in z-scores or freezing between the intervals with freezing and those without. Additionally, no correlation was observed between z-scores and freezing across all intervals or the intervals showing freezing. These results directly contrast with those of trace conditioning. This may be explained by a mechanistic difference between freezing in delayed and trace conditioning; that is, the former may predominantly involve an adaptive component, whereas the latter may involve more cognitive control [25,36]. Consistently, studies on human and mouse trace conditioning have suggested that attention or awareness plays a role in the process [28,37]. Similarly, in delayed non-matching to sample tasks, which also involve a delayed period, choice behavior replaces freezing, and a broader range of brain regions, including those engaged in trace conditioning, have been reported to participate [38–40].

For the two groups mentioned above (trace and delayed conditioning groups), there was no correlation between the z-score of individual neurons and percent freezing during the CS presentation period or the ITI period, whether considering only the periods when freezing was exhibited (S5 Fig) or considering all CS presentation or ITI periods (S6 Fig). Additionally, no correlation was observed between z-score and head velocity during the trace intervals (the hypothetical trace intervals for delayed conditioning retrieval group) where freezing was not exhibited across the two groups (Figs 3 and 4), which is consistent with our recent research concluding that ‘online neurons’ are unrelated to motor activity [5].

## Discussion

In this study, we identified a subset of rsCla neurons exhibiting characteristics of claustral ‘online neurons’ during trace conditioning, where freezing behavior was monitored in response to tone CS. Since we used tone CS and freezing behavior in the present study, this modality of CS and monitored behavior contrast with our recent study using light CS and delayed escape behavior [5]. We found a positive correlation between the z-scores of individual neurons and the percent freezing during the trace interval in the retrieval phases of the trace conditioning group, specifically when freezing was observed. However, the average z-scores between trace intervals with and without freezing were not significantly different. These findings suggest that while persistent neuronal activity is associated with freezing behavior during the trace interval, it does not directly cause the freezing. Instead, it may serve to modulate or influence freezing behavior during this period, but this conclusion requires confirmation through future experiments to determine whether inhibition of rsCla neurons attenuates freezing during the trace interval. In contrast, although a persistently enhanced activity was also observed after CS presentation during the retrieval phase in delayed conditioning, there was no correlation between z-scores of individual neurons and percent freezing during the hypothetical trace interval. This suggests that freezing behavior in trace conditioning differs from that in delayed conditioning in terms of regulatory mechanisms. Nevertheless, our data is consistent with the prediction that ‘online neurons’ exhibit activity that is independent of both the type of threat stimulus and the resulting behavior.

As shown in Fig 3, the enhanced activity of rsCla neurons persisted after the third CS during the retrieval phase of trace conditioning, spanning the CS, trace, and ITI periods. However, only the trace interval exhibited a significant correlation with freezing behavior. This finding supports the proposal that a subset of rsCla neurons encodes an online state; that is, the brain stays in a ready state for potential threats throughout the CS and ITI periods, with a particular bias towards predicting the US during the trace period. Previous studies suggest that the prefrontal cortex exhibits persistent activity during the trace period [30–32], whereas the hippocampus does not sustain persistent activity but instead shows specific learning responses to the CS and US [31,41,42]. This supports the hypothesis that the prefrontal cortex stores information about the CS in working memory form. For the hippocampus, it is suggested that it encodes the timing of the CS and US. Nonetheless, the claustrum is interconnected with nearly all other brain areas, suggesting that it contributes to bind neural traces dispersed across these brain regions into a coherent mental trace. In other words, assuming that information related to the CS is encoded in various regions of the brain and maintained as discrete working memory traces, inputs from these traces could potentially be integrated via the rsCla. In this context, the persistent activity in the rsCla could serve as an integrative maintainer of working memory traces distributed across the brain. Such a proposition is consistent with the hypothesis that rsCla neurons, defined as online neurons, play a role in maintaining the brain in an online state in response to specific threat stimuli.

Intriguingly, recent findings suggest that the claustrum plays a pivotal role in working memory, aligning well with our previous research [43]. Similarly, in our recent study [5], we observed that transient inhibition of persistent activity in the claustrum leads to irreversible suppression, providing evidence that recurrent or reentrant activity underlies claustral persistent activity. Supporting this notion, previous studies have demonstrated that the claustrum is interconnected with various brain regions [44] and exhibits excitatory connectivity among its own neurons [45]. Taken together, we propose a scenario in which the claustrum receives inputs from working memory traces across brain regions, integrates and maintains this information within itself, and subsequently provides feedback to these regions—a process that may iteratively repeat. The claustrum’s online neurons are hypothesized to play an active role in this mechanism. In conclusion, we predict that claustral online neurons facilitate the dynamic integration of working memory traces from diverse brain regions, enabling the coordinated processing and utilization of information across the brain.

The difference in the relationship between rsCla neuron activity and percent freezing during the trace interval in trace versus delayed conditioning can be explained by cognitive control in trace conditioning (but not exclusively) versus adaptive responses in delayed conditioning. Previous studies have indicated that freezing during the trace interval in trace conditioning is under cognitive control [31,36,46]. In contrast, freezing in delayed conditioning is well-known to be more adaptive, involving subcortical brain regions, particularly the amygdala [36,47]. Therefore, it is plausible that claustral ‘online neurons’, which are suggested to be involved in higher cognitive functions [5], are more likely to be associated with freezing behavior in trace conditioning where cognitive control is possible, whereas they would not be involved in freezing behavior in delayed conditioning.

In our recent study [5], the light CS, which was associated with the US through delayed conditioning, was used to elicit the delayed escape behavior. This is comparable to the CS presentation during the retrieval phase of delayed conditioning in the current study. The difference lies in the presence of an escape route in the delayed escape behavior, whereas no escape route was available in delayed conditioning. In this context, the persistently enhanced activity observed after the CS presentation with delayed conditioning in the current study supports the conclusion from the delayed escape behavior study; that is, the persistently enhanced activity of rsCla neurons serves to keep the brain in an online state in response to a threat stimulus, rather than encoding the motivation or intention to escape. In the case of delayed conditioning, where the monitored behavior is freezing, it is challenging to measure the impact of rsCla ‘online neurons’ since they might preferably influence behaviors under cognitive control. That is, while ‘online neurons’ in the rsCla would be active during the retrieval phase of delayed conditioning, there would be no measurable behaviors to observe their impact. Future research should explore whether rsCla ‘online neurons’ can be recruited by rewarding stimuli as well as threats. Another difference concerns the termination of persistently enhanced activity. During the delayed escape test, we observed that persistently enhanced activity returned to baseline after the escape, consistent with the possibility that the potential threat had been resolved via the escape. However, in trace or delayed conditioning, the enhanced activity persisted even after the trace interval or CS presentation. This suggests that the online state continues to be maintained in these paradigms, possibly because the potential threat remains unresolved.

If extended to human studies, it would be particularly intriguing to investigate the mechanisms through which the repertoire of related memories is connected via the claustrum [51,52]. Human studies suggest that suppressing specific memory traces can protect these memories and support further learning [52]. Conversely, the failure of such suppression may result in interference between existing and newly formed memories, leading to cognitive difficulties [52]. Indeed, artificial neural networks that lack a mechanism to suppress and render existing memories latent have been reported to suffer catastrophic outcomes when tasked with multiple learning processes. Conversely, the activation of specific memory traces is known to play a pivotal role in memory retrieval, highlighting the importance of selective suppression and activation of memory traces as central processes in information processing [52]. This underscores the potential necessity of a central control tower capable of modulating the inhibition or excitation of different brain regions—a role for which the claustrum is a strong candidate. The outputs of the claustrum are known to vary across brain regions and may be either excitatory or inhibitory [48]. In this context, the claustrum may support sequential learning or facilitate the retrieval of specific memories by modulating working memory traces in a region-specific manner through excitation or inhibition. If such functions are disrupted, they could lead to pathological outcomes, such as the excessive enhancement of memory (e.g., post-traumatic stress disorder) or impairments in memory formation and retrieval (e.g., schizophrenia) [52].

Previous tracing results indicate that the claustrum receives inputs from brain regions involved in maintaining the brain’s online state for meaningful stimuli, such as the basolateral amygdala (emotional information) [49–51], prefrontal cortex (planning) [52,53], and orbitofrontal cortex (decision-making) [54,55]. Also, it has been suggested that the claustrum has functional roles in spatial processing based on inputs from hippocampal regions [21–23], which could be utilized for flexible computation of behavioral trajectories [56,57]. These aforementioned neural inputs may be integrated into the claustrum and re-distributed to its downstream neural networks, and the integrated information may linger via the re-entrant network involving the claustrum. This prediction is consistent with the presence of persistent activity that may be re-entrant and/or recurrent as shown in our recent study [5], reminiscent of “global ignition” [58] that is associated with high-level cognition in non-human primates. For future studies, brain-wide recordings will be required to explore the role of claustral ‘online neurons’.

## Methods

### Animals

Male Long Evans rats, eight weeks old (Japan SLC Inc.), were housed in pairs for 5–11 days before the start of experiments under a 12-hour reversed light/dark cycle (lights off at 9:00 a.m.) with free access to food and water. All behavioral tests were carried out during the dark phase of the cycle. Each animal was handled for 1 day prior to the experiments. Twelve rats were used for the trace conditioning experiment, and 17 rats were used for the delayed conditioning experiment, with different rats used for each experiment (S1C Fig). Trace conditioning and delayed conditioning experiments were conducted in a non-blind manner. The Institutional Animal Care and Use Committee (IACUC) of Seoul National University approved all experimental protocols, and we conducted all procedures in accordance with the guidelines for the care and use of laboratory animals provided by Seoul National University.

### Trace and delayed conditioning procedures

#### Day 1: habituation.

We placed 8-week-old male Long Evans rats in a rectangular chamber, illuminated by 1W white LED light. Before each session, we sanitized the chamber with 70% ethanol. During the initial session, we conducted tone habituation by presenting five 30-second pip tones (CS; 2.8 kHz, 85 dB) after a 4-minute baseline. The average ITI was set at 90 seconds.

#### Day 2: acquisition phase of conditioning.

The same rectangular context was used as on Day 1 and sanitized with 70% ethanol. For delayed conditioning, we paired a 30-second pip tone (CS; 2.8 kHz, 85 dB) with a co-terminated 0.7 mA, 1-second electric foot shock (US) after a 120-second baseline. The CS and US were paired directly, with an average ITI of 157.5 seconds (from the end of the trace interval to the onset of the next CS). In trace conditioning, we introduced a 10-second gap between the end of the CS and the onset of the US. A total of 7 CS and US presentations were conducted for fear conditioning acquisition. Rats were removed from the chamber 1 minute after the last US presentation.

#### Day 3: retrieval phase of conditioning.

The retrieval session took place in a cylindrical chamber measuring 27 ×  27 ×  30 cm, illuminated with a 1W red LED light (625 nm wavelength). Prior to the session, we sanitized the chamber with 70% ethanol and subsequently treated it with 1% acetic acid. The session began with a 4-minute baseline, followed by five presentations of the CS without the US, with an average ITI of 65 seconds (from the end of the trace or hypothetical trace interval to the onset of the next CS). During these sessions, we monitored and measured freezing behavior. Additionally, we videotaped all sessions for post-hoc measurement of freezing. Freezing was defined as the absence of all movement, except for respiration, sustained for at least one second and measured by expert observer. Head velocity was scored by labeling both ears of rats in all recorded frames, and the speed of the midpoint between the two ears was calculated using MATLAB.

### In vivo single-unit recordings

We anesthetized the rats with an intraperitoneal injection of sodium pentobarbital (50 mg/kg) and maintained anesthesia with isoflurane (1–1.5%) in O2. We mounted the rats on a stereotaxic apparatus (Stoelting Co.) and implanted fixed-wire electrodes bilaterally into the rsCla (AP + 3.35/ML ±  2.10/DV-4.50). We also implanted a ground wire in the cerebellum. The electrodes, consisting of eight individually insulated nichrome microwires (50 μm outer diameter, impedance 1–3 MΩ; California Fine Wire) contained in a stainless-steel guide cannula, were affixed to the skull with screws using Poly-F zinc polycarboxylate cement (Konstanz), vertex self-curing (vertex-dental, Zeist), and bond (Loctite 411, Henkel). We allowed the rats a week to recover before they underwent experimental procedures.

We acquired neural activity using a Plexon MAP system and performed data analysis with Offline Sorter (Plexon). We plotted all waveforms obtained from a channel in the principal component space, and clusters consisting of similar waveforms were defined both automatically and manually. Single-unit isolation was graded using two statistical parameters: J3 and the Davies-Bouldin validity metric (DB). A high J3 and low DB value indicated a compact, well-separated unit cluster, and neurons with a low grade were discarded.

We defined ‘exploratory neurons’ as neurons whose firings were significantly correlated with exploratory behaviors, such as rearing and context boundary investigation. To identify such ‘exploratory neurons’ in each rat, we monitored exploratory behaviors with a 0.1 s-binned time series before the presentation of the first CS in the retrieval phase. Each bin was marked in a binary fashion as labeled (exploratory behaviors observed) or non-labeled. Rearing was defined as the behavior where the rat stood only on its hind legs, and context boundary investigation as the behavior where the rat dug its nose into the boundary of the acquisition and retrieval chambers and sniffed. We calculated the firing rates within the 0.1 s bins for each neuron and compared the firing rates between labeled and non-labeled bins using a rank test (Mann–Whitney test). When the difference was statistically significant (p < 0.01), we classified the neuron as an ‘exploratory neuron’.

We conducted clustering by aggregating all neurons measured across the two groups (trace conditioning retrieval, delayed conditioning retrieval). We categorized the firing data from recorded neurons into sixteen epochs: baseline, cue × 5, interval × 5, and ITI × 5 epochs. Unlike the other ITIs, the last of the five ITIs was maintained for a shorter duration of 15 seconds. The z-score transformation involved normalizing the neural activity during each epoch to the baseline (20-second period before first CS onset, binned at 1 second). In cases where there was no firing within the initial 20-second period, we extended the baseline until firing was detected, either spanning the whole pre-CS period or the entire session. We subjected all neurons (n  =  173) to three-dimensional dimensionality reduction using t-SNE, utilizing z-scores from the five epochs excluding the baseline. t-SNE (t-distributed stochastic neighbor embedding) is one of the methods for dimensionality reduction of high-dimensional data, along with PCA and UMAP. A unique feature of t-SNE is its non-linear dimensionality reduction approach. The t-SNE algorithm calculates the similarity of points in high-dimensional space and their corresponding similarities in low-dimensional space. This allows it to reduce the dimensionality while preserving the distance distribution of data points as much as possible in the lower-dimensional space. We grouped neurons within the t-SNE space using the K-means clustering method. To determine the optimal number of clusters for K-means clustering in an unbiased manner, we employed the gap statistic[5,33,35]. For each identified cluster, we plotted the averaged z-scores (1 second binned) of the clustered neurons against the recording period.

We calculated the average z-scores for single neurons recorded from a specific rat during the cue, trace interval, and ITI periods, and measured the rat’s average percent freezing during each of these periods. The z-scores and percent freezing values for each period were then obtained for all rats in the specific experimental group. These values were plotted for each period, and the correlation coefficient and statistical significance were determined. As each rat received 5 CS presentations, 5 data points for the z-scores and 1 freezing value per rat were plotted.

### Histology

At the end of the experiments, we anesthetized the rats with urethane (1 g/kg, i.p.) and made electrolytic lesions by passing current (10 mA, 5–20 seconds) through the recording microwires from which discrete units were identified, to determine the location of the microwires. We then perfused the animals transcardially with 0.9% saline solution followed by 10% buffered formalin. The brains were removed and post-fixed overnight. Coronal sections (90 µm thick) were obtained using a vibroslicer (NVSL; World Precision Instruments, Sarasota, FL) and stained with cresyl violet. The placement of the recording microwires was examined under a light microscope. Images of the slices were captured using the ZEISS Axio Scan.Z1 with a 10X objective. Finally, the location of the microwires was determined as previously described [5].

### Statistics

Statistical analyses were conducted using Prism9 (GraphPad) and MATLAB (MathWorks). Before performing statistical comparisons, we first conducted D’Agostino–Pearson omnibus normality tests or Kolmogorov-Smirnov lognormality tests, as appropriate. If all groups passed the normality tests (p > 0.05), parametric statistics (two-tailed paired or unpaired t-test, one-way ANOVA with subsequent Holm–Sidak’s multiple comparisons test, mixed-effects analysis, or Pearson correlation, as appropriate) were used. If at least one group did not pass the normality test, we used non-parametric statistics [59,60] (two-tailed Wilcoxon signed-rank or Mann–Whitney test, Kruskal–Wallis or Friedman test with subsequent Dunn’s multiple comparisons test, multiple Mann–Whitney tests with subsequent Holm–Sidak method, or Spearman correlation, as appropriate). Data are expressed as mean ±  standard error of the mean (s.e.m.). Statistical significance was set at * p  <  0.05, **p  <  0.01, ***p  <  0.001, and ****p  <  0.0001. Exceptionally, the statistical analysis comparing multiple mean values shown in Fig 3 employed the Friedman test followed by multiple comparisons using the False Discovery Rate (FDR) method by Benjamini and Hochberg.

## Supporting information

S1 Fig Clustering parameters. (A) Results from the gap statistics after t-SNE dimensional reduction. According to the gap statistics, when the gap statistic value for a clustering number is lower than the value for the next higher clustering number minus its standard error, this indicates a significant clustering number. The smallest significant clustering number is then chosen. In our case, this value was determined to be 3. (B) Clustering result when the recorded neurons were clustered into two groups. Dividing the clusters into two did not significantly alter the composition of the neurons in the persistent neuron group, compared to when the clusters were divided into three. (C) Number of exploratory and non-exploratory neurons, in each cluster. It also shows the number of rats used for each group.
(TIF)

S2 Fig Clustering of only non-exploratory claustral neurons based on behavioral epochs. (A) Distribution of clustered, non-exploratory neurons (n = 102) in t-SNE space. (B) Z scored firing rates of neurons within each cluster. (C) The mean *Z* score values of non-exploratory neurons in cluster 1 of the trace conditioning retrieval group (n =  12) were found to change as the behavioral sessions progressed (**p =  0.0014; Friedman test followed by FDR by Benjamini and Hochberg, Q <  0.05 are marked with a star). These neurons are all included among the 14 neurons shown in Fig 3B. (D) The mean *Z* score values of non-exploratory neurons in cluster 1 of the delayed conditioning retrieval group (n =  14) were found to change as the behavioral sessions progressed (****p <  0.0001; Friedman test followed by FDR by Benjamini and Hochberg, Q <  0.05 are marked with a star). These neurons are all included among the 17 neurons shown in Fig 4B. Based on these results, clustering performed only on non-exploratory neurons produced very similar outcomes to the clustering performed on all neurons.
(TIF)

S1 Table Firing rates of non-exploratory neurons by cluster. (PPTX)

S3 Fig Classified putative pyramidal neurons and putative interneurons. (A) plot each neuron’s basal firing rate, area under peak, and spike half-width in three-dimensional space to classify pyramidal neurons and interneurons. (B) The proportion of classified neurons overall and their characteristics. (C) The proportion and characteristics of classified putative pyramidal neurons and putative interneurons among non-exploratory neurons in cluster 1 during trace conditioning retrieval. (D) Same data for delayed conditioning retrieval. (E) The number of neurons categorized as putative pyramidal neurons, putative interneurons, and their classification based on experimental clusters and behavioral relevance. (F) Same t-SNE graph as Fig 2A, but labelled putative pyramidal neurons and interneurons.
(TIF)

S4 Fig Heatmap of non-exploratory neurons in cluster 1. (A) Trace conditioning retrieval (B) Delayed conditioning retrieval
(TIF)

S5 Fig Correlation between the average z-scores during the cue, interval, and ITI (inter-trial interval) phases of non-exploratory neurons in cluster 1 and freezing during those periods. This figure includes only the periods where freezing occurred. (TIF)

S6 Fig Correlation between the average z-scores during the cue, interval, and ITI phases of non-exploratory neurons in cluster 1 and freezing during those periods. This figure includes all periods, irrespective of whether freezing occurred within each period. (TIF)

S7 Fig Activity patterns of non-exploratory neurons in clusters 2 and 3, and their correlation with freezing. (A) The mean z-score values of non-exploratory neurons in cluster 2 of the trace conditioning retrieval group (n =  11) were found to change as the behavioral sessions progressed (Friedman test, **p =  0.0091; FDR by Benjamini and Hochberg, Q < 0.05 marked). (B) Average z-scores during trace conditioning retrieval intervals with and without freezing (n = 15, 5 respectively; p =  0.3486 via Mann–Whitney tests). (C) Correlation between freezing rates and z-scores of non-exploratory cluster2 neuron during trace conditioning retrieval intervals (n =  20; Spearman correlation r =  -0.2323, p =  0.2168). This figure includes all periods, irrespective of whether freezing occurred within each period. (D) The mean z-score values of non-exploratory neurons in cluster 3 of the trace conditioning retrieval group (n =  16) were found to change as the behavioral sessions progressed (Friedman test, ****p =  0.0006; FDR by Benjamini and Hochberg, Q < 0.05 marked). (E) Average Z-scores during trace conditioning retrieval intervals with and without freezing (n = 29, 11 respectively; * p =  0.0110 via Mann–Whitney tests). (F) Correlation between freezing rates and z-scores of non-exploratory cluster3 neuron during trace conditioning retrieval intervals (n =  40; Spearman correlation r =  -0.4152, **p =  0.0077). This figure includes all periods, irrespective of whether freezing occurred within each period. (G) The mean z-score values of non-exploratory neurons in cluster 2 of the delayed conditioning retrieval group (n =  24) were found to change as the behavioral sessions progressed (Friedman test, ****p <  0.0001; FDR by Benjamini and Hochberg, Q < 0.05 marked). (H) Average Z-scores during delayed conditioning retrieval intervals with and without freezing (n = 36, 14 respectively; ****p <  0.0001 via Mann–Whitney tests). (I) Correlation between freezing rates and Z-scores of non-exploratory cluster2 neuron during delayed conditioning retrieval intervals (n =  50; Spearman correlation r =  -0.5921, ****p <  0.0001). This figure includes all periods, irrespective of whether freezing occurred within each period. (J) The mean z-score values of non-exploratory neurons in cluster 3 of the delayed conditioning retrieval group (n =  20) were found to change as the behavioral sessions progressed (Friedman test, **p =  0.0037; FDR by Benjamini and Hochberg, Q < 0.05 marked). (K) Average Z-scores during delayed conditioning retrieval intervals with and without freezing (n = 40, 10 respectively; p =  0.0791 via Mann–Whitney tests). (L) Correlation between freezing rates and Z-scores of non-exploratory cluster3 neuron during delayed conditioning retrieval intervals (n =  50; Spearman correlation r =  0.1035, p =  0.4744). This figure includes all periods, irrespective of whether freezing occurred within each period.
(TIF)

S8 Fig Histological verification of *in vivo* single unit recordings in the rsCla. (A) Electrode tip placements in the trace conditioning group (black circle, n  =  11). (B) Electrode tip placements in the delayed conditioning group (black circle, n  =  17). The target regions of rsCla (red dashed line) are defined as in Han et al. (2024). The atlas was adapted from Paxinos and Watson (2014).
(TIF)
