# Supplementary material for: Single-unit activity in the anterior claustrum during memory retrieval after trace fear conditioning
Source: PLoS One. 2025 Feb 11;20(2):e0318307. doi: 10.1371/journal.pone.0318307 (PMC11813112; doi:10.1371/journal.pone.0318307)
Supplement: S1 Table — (PPTX) [file pone.0318307.s003.pptx]

## Slide 1
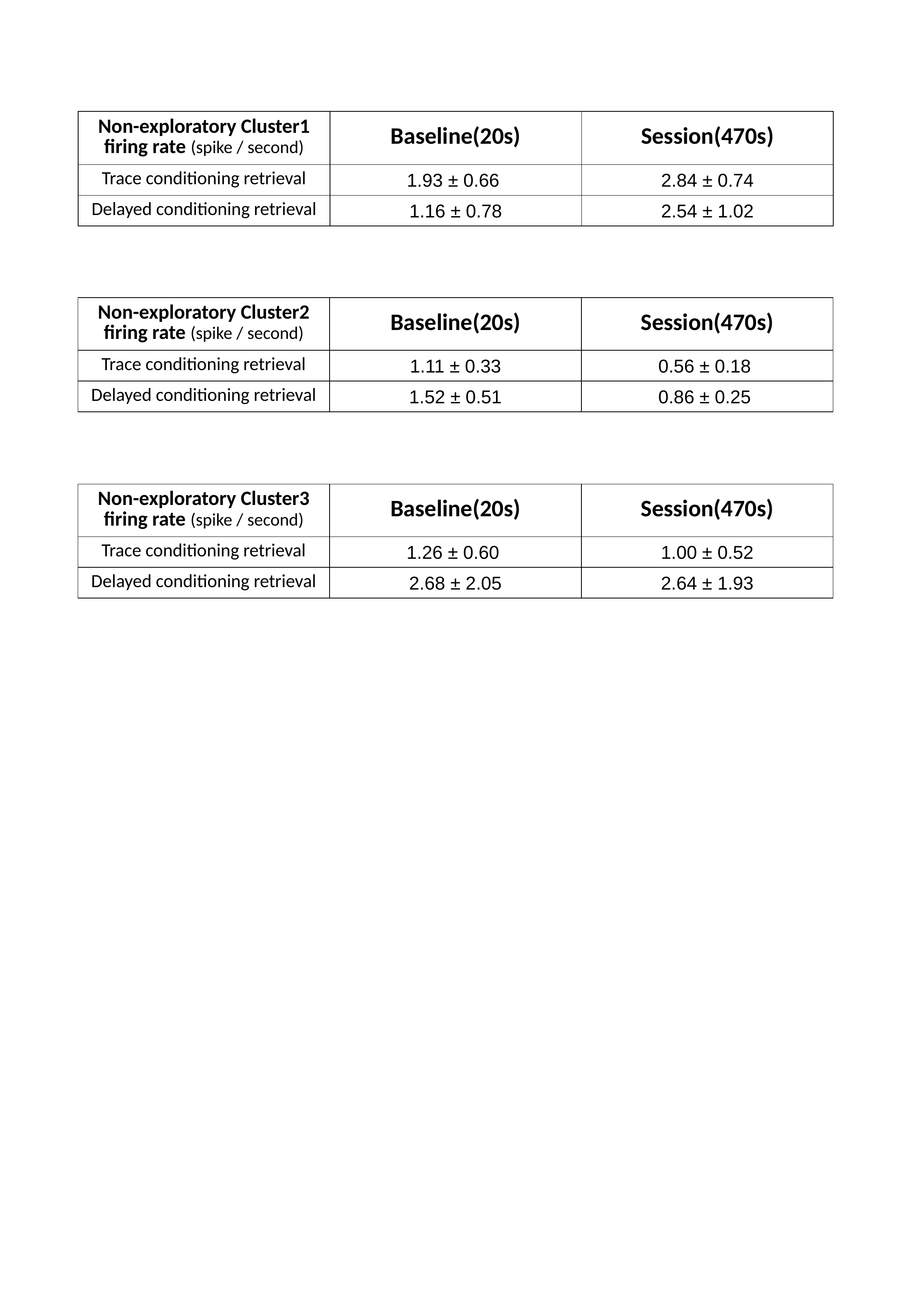

| Non-exploratory Cluster1 firing rate (spike / second) | Baseline(20s) | Session(470s) |
| --- | --- | --- |
| Trace conditioning retrieval | 1.93 ± 0.66 | 2.84 ± 0.74 |
| Delayed conditioning retrieval | 1.16 ± 0.78 | 2.54 ± 1.02 |
| Non-exploratory Cluster2 firing rate (spike / second) | Baseline(20s) | Session(470s) |
| --- | --- | --- |
| Trace conditioning retrieval | 1.11 ± 0.33 | 0.56 ± 0.18 |
| Delayed conditioning retrieval | 1.52 ± 0.51 | 0.86 ± 0.25 |
| Non-exploratory Cluster3 firing rate (spike / second) | Baseline(20s) | Session(470s) |
| --- | --- | --- |
| Trace conditioning retrieval | 1.26 ± 0.60 | 1.00 ± 0.52 |
| Delayed conditioning retrieval | 2.68 ± 2.05 | 2.64 ± 1.93 |
